# Genistein Modulated Lipid Metabolism, Hepatic PPARγ, and Adiponectin Expression in Bilateral Ovariectomized Rats with Nonalcoholic Steatohepatitis (NASH)

**DOI:** 10.3390/antiox10010024

**Published:** 2020-12-29

**Authors:** Sudaporn Pummoung, Duangporn Werawatganon, Maneerat Chayanupatkul, Naruemon Klaikeaw, Prasong Siriviriyakul

**Affiliations:** 1 Alternative and Complementary Medicine for Gastrointestinal and Liver Diseases Research Unit, Department of Physiology, Faculty of Medicine, Chulalongkorn University, Bangkok 10330, Thailand; yhinahs@hotmail.com (S.P.); maneeratc@gmail.com (M.C.); fmedpsr@gmail.com (P.S.); 2 Department of Pathology, Faculty of Medicine, Chulalongkorn University, Bangkok 10330, Thailand; wnaruemon@gmail.com

**Keywords:** non-alcoholic steatohepatitis, genistein, ovariectomized, apoptosis, PPARγ, adiponectin

## Abstract

The aim of this study was to evaluate the protective effects of genistein on lipid accumulation and apoptosis in estrogen deficient rats with NASH. Female Sprague–Dawley rats (n = 48) were divided into ovariectomized (OVX) and non-OVX groups. Each group was then sub-divided into 3 subgroups; control, NASH (rats fed with a high-fat, high-fructose (HFHF) diet), and NASH+Gen (rats fed with HFHF diet plus daily genistein at 16 mg/kg BW). Results showed that HFHF diet induced liver fat accumulation in both non-OVX and OVX rats, which was evidenced by hepatic steatosis on liver pathology and increased hepatic free fatty acid (FFA) and triglyceride levels. Hepatic fat accumulation was significantly more severe in NASH rats with OVX than non-OVX. Hepatocyte apoptosis was more severe in NASH groups compared with that in control groups. Genistein administration significantly improved histopathology of NASH in both non-OVX and OVX rats and attenuated hepatic lipid accumulation, oxidative stress, and hepatocyte apoptosis. Genistein also down-regulated PPARγ and up-regulated adiponectin expression. In summary, NASH could be worsened by estrogen deficiency, indicating the protective action of estrogen on NASH. Genistein administration alleviated hepatic steatosis and apoptosis through the down-regulation of PPARγ and up-regulation of adiponectin expression.

## 1. Introduction

Nonalcoholic steatohepatitis (NASH) is well-recognized as a manifestation of metabolic syndrome. Because of a strong correlation between metabolic syndrome and NASH [1], the increase in obesity prevalence makes NASH a major cause of chronic liver diseases. A 170% increase in non-alcoholic fatty liver disease (NAFLD) prevalence over the past ten years (2004–2013) has recently been reported. Histological evidence of NASH was seen in 6–13% of all NAFLD patients [2]. Some patients may progress from NASH to cirrhosis, which increases the risk of hepatocellular carcinoma [3].

The two-hit hypothesis has been proposed in the pathogenesis of NASH, with the first hit being a dysfunction of lipid metabolism leading to lipid accumulation in the liver. Lipid accumulation subsequently induces liver inflammation and hepatocyte damage as a second hit [4]. NASH development involves several processes and is attributed to many factors, such as metabolic syndrome, insulin resistance, adipokines, gut dysbiosis, and genetic susceptibility. 

Estrogen is the primary female sex hormone, and it possesses many biological functions, such as lipid and glucose metabolism, cell differentiation, and inflammatory modulation [5]. Prior studies suggest a possible link between estrogen deficiency and NASH. Estrogen deficiency is associated with the alteration of lipid metabolism and hyperlipidemia [6]. Being on the same high-fat, high-cholesterol diet, ovariectomized mice manifested a more severe form of NASH than sham-operated mice [7]. Furthermore, the incidence of NASH in women younger than 50 years old was 1.8-fold less than that in men, while the incidence rates were similar between men and post-menopausal women [8]. In this study, we aimed to determine the effect of estrogen deficiency in NASH development and its molecular mechanisms.

Currently, there are no FDA-approved medications for the treatment of NASH. Hormone replacement therapy may reduce the risk of NASH in post-menopausal women but its use is associated with the increased risk of certain cancers. Phytoestrogen such genistein might be a reasonable alternative treatment for NASH in this setting. Genistein, an isoflavone found in soybean, has a nearly identical structure to estrogen and its actions resemble those of estrogen. Previous studies demonstrated that genistein reduced the levels of tumor necrosis factor-alpha (TNF-α), interleukin 6 (IL-6), and lipid peroxidation in rats with NASH [9]. Genistein also decreased hepatic fatty acid synthase activity [10] and increased the expression of peroxisome proliferator-activated receptor-gamma (PPARγ) [11] in NASH. The effect of genistein on PPARγ, however, remains controversial, as another animal study using apolipoprotein E (ApoE) knockout mice has shown that genistein attenuated hepatic PPARγ expression level in NASH [12]. Moreover, whether genistein affects adipokine levels such as adiponectin is also a matter of debate. The objectives of the current study were to determine the benefits of genistein supplement on NASH induced by a high-fat, high-fructose (HFHF) diet in the setting of estrogen deficiency and its effects on lipid metabolism, oxidative stress, hepatocyte apoptosis, hepatic PPARγ expression, and adiponectin levels.

## 2. Materials and Methods

### 2.1. Animals and Experimental Protocols

All animal procedures in this study complied with the recommendations and guidelines for the use of experimental animals by the National Research Council of Thailand and the protocol was approved by the Animal Care and Use Committee, Faculty of Medicine, Chulalongkorn University (the permission No. 19/2559).

Initially, 48 4-week-old female Sprague–Dawley rats were housed under standard conditions and fed with a standard diet. After one week of acclimatization, rats were randomly assigned into 2 major groups: non-ovariectomized (non-OVX) and ovariectomized (OVX). Then, rats in both groups were divided into 3 subgroups (8 rats per group): rats fed with a standard diet (control group), rats fed with a HFHF diet (NASH group), and rats fed with a HFHF diet with daily oral gavage of 16 mg/kg body weight of genistein dissolved in 1 mL of 0.1% dimethyl sulfoxide (NASH+Gen group). Rats in control and NASH groups received 1 mL of 0.1% dimethyl sulfoxide once daily as a vehicle control. OVX rats underwent bilateral ovariectomy with a double dorsolateral approach under anesthesia. Vaginal smear was performed for 5 consecutive days to confirm that the ovaries were completely removed. At the end of the experimental period (8 weeks), liver tissues and blood samples were collected for further analysis. 

In control groups, rats were fed with a standard diet provided by the Perfect Companion Group Co., Ltd., Thailand. The standard diet included 7% fat, 47% carbohydrates, and 27% protein. A HFHF diet was given to induce NASH pathology in the NASH groups. The formula of the HFHF diet was modified from Pickens MK [13], which consisted of 55% fat from vegetable oil, 35% carbohydrate from flour with 20% fructose, and 10% protein from egg albumin. All diets were fed ad libitum.

### 2.2. Liver Tissue Analyses

#### 2.2.1. Hepatic Histopathology

Liver tissues were fixed in 10% formaldehyde for 24–48 h before tissue embedding. The embedded livers were cut into 3-µm-thick sections followed by deparaffinization. Subsequently, sections were stained with hematoxylin and eosin. All stained samples were analyzed and graded for the degree of steatosis, necroinflammation, and hepatocellular ballooning, following the criteria of Brunt [14], by an experienced pathologist who was blinded to the experimental group. 

#### 2.2.2. Hepatic Lipid Accumulation; Oil Red O (ORO) Stain, Free Fatty Acid (FFA) and Triglyceride (TG) Level 

##### Oil Red O Stain

First, the −80 °C frozen livers were thawed on ice, then 0.5 cm samples were embedded with Tissue-Tek^®^ OCT compound (Sakura Finetek, Torrance, CA) on cryomolds. After consolidation, frozen-embedded liver tissue was further cut into 5-µm-thick sections by cryostat microtome at −20 °C and pasted on adhesive microscope slides (Matsunami Platinum PRO, Tarjan Scientific, Ringwood, Victoria, Australia). Immediately, liver sections were stained with Oil Red O (ORO) solution for 3 min. Subsequently, sections were counterstained with hematoxylin and mounted with fluorescence mounting medium (Dako North America Inc, Carpinteria, CA, USA). Photographic analysis with regular light microscope was performed within 24 h. Twenty fields of each liver section at 40× magnification were used to analyze the area that was occupied by lipid droplets [15] with Image-Pro plus software (Media Cybernetics, Rockville, MD, USA) and expressed as percentage of ORO-positive area.

##### Colorimetry for Free Fatty Acid and Triglyceride Analysis

According to the manufacturer’s protocol, lipid was extracted from the liver tissue with a lipid extraction kit (BioVision, Inc., Milpitas, CA, USA). The extracted lipid was then suspended in 50 µL of lipid suspension buffer and sonicated for 15–20 min at 37 °C before quantifying the level of FFA (FFA quantification colorimetric kit, BioVision Inc., Milpitas, CA, USA) and TG (Triglyceride quantification colorimetric kit, BioVision Inc., Milpitas, CA, USA) by colorimetric assay. Hepatic FFA and TG levels were expressed as nmol/mg of tissue.

### 2.3. Hepatic Lipid Peroxidation by Thiobarbituric Acid Reactive (TBAR) Assay

Liver tissue (0.1 g) was homogenized on ice for 30 min in 1 mL of RIPA buffer (Cell Signaling Technology^®^, Inc., Danvers, MA, USA) with protease inhibitor cocktails (Sigma-Aldrich, DS, Germany). Bicinchoninic acid (BCA) assay was performed to determine protein concentration in the supernatant by BCA protein assay kit (Pierce^®^, Thermo scientific, Inc., Rockford, IL, USA).

Consequently, the degree of hepatic lipid peroxidation, or the malondialdehyde (MDA) level, was determined by calculating the production rate of thiobarbituric acid reactive substances (TBARS) with commercial assay kits (Cayman chemical, Ann Arbor, MI, USA). Briefly, supernatant was mixed with the solution containing 20% acetic acid, 0.8% thiobarbituric acid, and 8.1% sodium dodecyl sulfate. The solution was boiled for 1 h in 95 °C water bath and then centrifuged for 10 min at 1600 g. The absorbance of the supernatant fraction was measured at 532 nm wavelength and expressed in nmol/mg protein.

### 2.4. Hepatocyte Apoptosis by TUNEL Assay

TUNEL assay was performed according to the manufacturer’s instructions (Millipore, Temecula, CA, USA). Cells with dark brown nuclei were considered TUNEL positive cells and counted by the Aperio ImageScope software (Leica Biosystems Imaging, Inc., Baltimore, MD, USA). A total of 1000 hepatocytes were counted (100 cells/20xfield, 10 fields total). The result was expressed as the percentage of positive immunoreactive cells.

### 2.5. Hepatic Peroxisome Proliferator-Activated Receptor Gamma (PPARγ) and Adiponectin Protein Expression by Western Blot Analysis

The 60 µg of denatured protein was loaded to 10% sodium dodecyl sulfate polyacrylamide gel electrophoresis (SDS-PAGE), then was run in a buffer tank for 30 min with the initial voltage at 80 V. Voltage was then increased to 120V for 60 min. The separated proteins were transferred to polyvinylidene fluoride (PVDF) membrane by the semi-dry transfer method (Bio-Rad, Hercules, CA, USA). PVDF membrane that contained proteins of interest was placed in 5% non-fat dry milk in phosphate buffer saline with Tween 20 (PBST) at 4 °C overnight. Subsequently, PVDF membrane was incubated for 2 h at room temperature with PPARγ (Santa Cruz Biotechnology, Inc., Dallas, TX, USA; 1:400) or adiponectin primary antibodies (R&D system, Minneapolis, MN, USA; 1:500). After that, the membrane was washed for 5 min by Tris-buffer saline with Tween 20 (TBST) 3 times and incubated with a secondary antibody—conjugated horseradish peroxidase (HRP) (1:10,000) (goat anti-mouse for PPARγ, Santa Cruz Biotechnology, Inc., Dallas, TX, USA or donkey anti-goat for adiponectin, Abcam, Cambridge, MA, USA) for 1 h at a room temperature. The membrane was washed 3 times; 5 min each, with TBST before bands of target protein were visualized with enhanced chemiluminescence (ECL) western blotting system (ChemiDocTM Touch Imaging System, BioRad laboratories, Hercules, CA, USA) and normalized by β-actin using Image LabTM Software (BioRad laboratories, Hercules, CA, USA).

### 2.6. Statistical Analysis

The data were reported as mean ± standard deviation. One-way analysis of variance and least significant difference (LSD) post-hoc test were used to compare the mean difference among experimental groups. Descriptive statistics were used for histological examination. A *p* < 0.05 was considered statistically significant.

## 3. Results

### 3.1. Gross Liver Appearance and Histopathology

**Figure 1:**
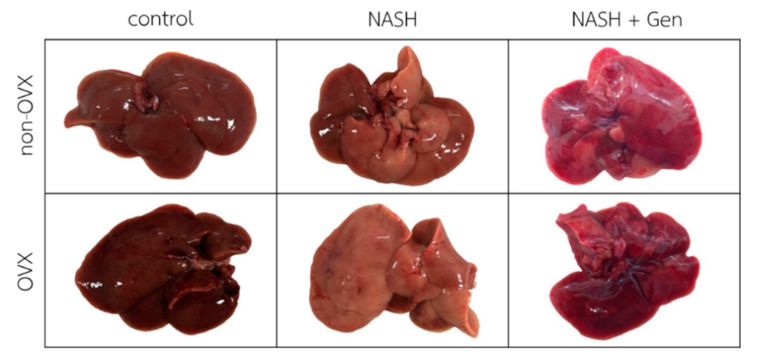
Gross liver appearance of rats in all experimental groups. The color of the liver is yellow-tinged and greasy in non-OVX and OVX with the high-fat, high-fructose (HFHF) diet groups. Livers in genistein groups were pink-red in color. Non-OVX: non-ovariectomized; OVX: ovariectomized; NASH: nonalcoholic steatohepatitis.

Gross liver appearance in rats fed with the HFHF diet was yellow-tinged in both non-OVX and OVX groups as compared with reddish-brown in rats fed with a normal diet. The yellow color was more pronounced in livers of OVX than non-OVX rats. Genistein at the concentration of 16 mg/kg body weight improved the gross appearance of the liver in both non-OVX and OVX groups (Figure 1).

**Figure 2:**
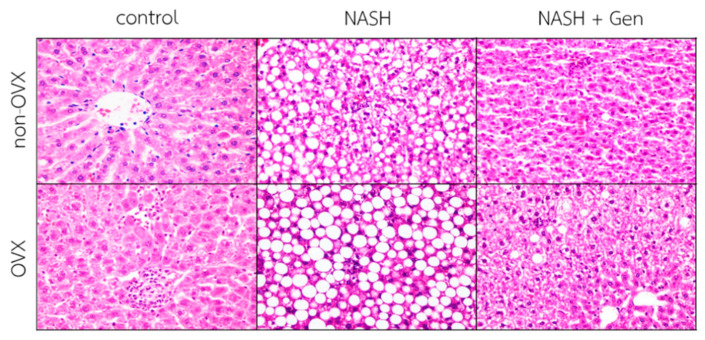
Hematoxylin–eosin (H&E) stain of liver sections. The degree of steatosis, lobular inflammation, and hepatocellular ballooning were the most severe in OVX rats with NASH. Genistein improved steatosis, lobular inflammation, and hepatocellular ballooning in both non-OVX and OVX rats with NASH. The original magnification is 400×. Non-OVX: non-ovariectomized; OVX: ovariectomized; NASH: nonalcoholic steatohepatitis.

Hematoxylin–eosin (H&E) stain of liver sections demonstrated that the HFHF diet induced the typical features of NASH, including steatosis, lobular inflammation, and ballooning of hepatocytes in both non-OVX and OVX rats. Additionally, liver injuries were more severe in OVX than non-OVX rats fed with the HFHF diet. Interestingly, OVX rats fed with a standard diet exhibited more lipid accumulation and lobular inflammation than the corresponding non-OVX rats. Genistein administration improved liver steatosis, lobular inflammation, and hepatocellular ballooning in both non-OVX and OVX rats with NASH (Figure 2).

**Figure 3:**
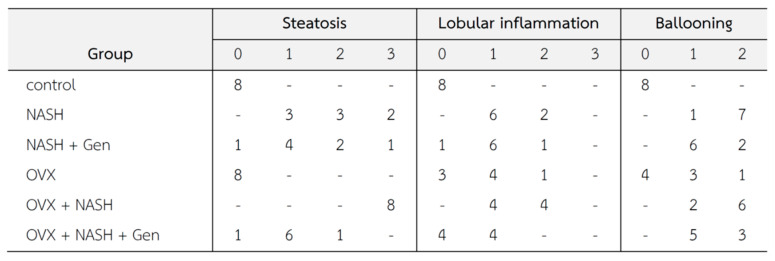
Changes in liver tissue (*n* = 8 in each group). Criteria of histopathological scores are as fellows [3]; Steatosis: 0 = no hepatocytes containing fat; 1 ≤ 33% of the hepatocytes containing fat; 2 = 33–66% of the hepatocytes containing fat; 3 = > 66% of the hepatocytes containing fat; Ballooning: 0 = no ballooning; 1 = few ballooning; 2 = many ballooning; Lobular inflammation: 0 = no inflammation and necrosis; 1 = mild zone 3 injury/inflammation; 2 = noticeable zone 3 injury/inflammation; 3 = severe zone 3 injury/inflammation.

Histopathological lesions were graded according to Brunt’s criteria [3] by an experienced histopathologist. Steatosis, lobular inflammation, and hepatocellular ballooning scores were higher in non-OVX and OVX rats fed with a HFHF diet when compared with control groups. Noticeably, overall histological scores of NASH were higher in OVX than in non-OVX rats fed with a HFHF diet. The administration of genistein reduced the NASH score in both non-OVX and OVX rats with NASH when compared with non-treatment groups (Figure 3).

### 3.2. Hepatic Lipid Peroxidation Level

**Figure 4:**
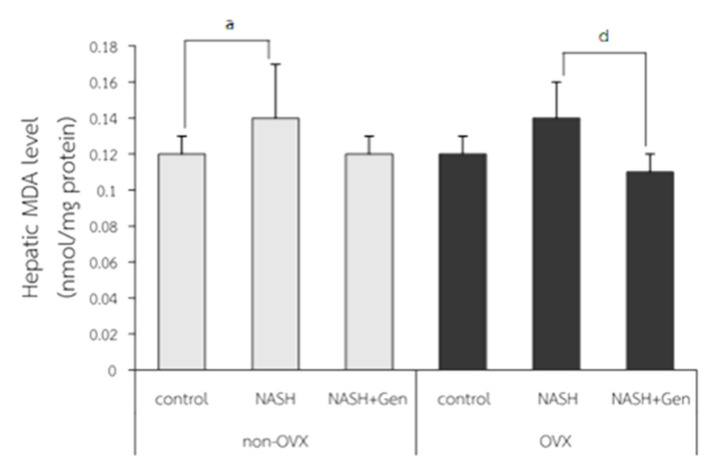
Malondialdehyde (MDA) level measured by TBARS assay. a: *p* < 0.05 comparison between control and NASH groups of non-OVX rats. d: *p* < 0.05 comparison between NASH and NASH+Gen groups of OVX rats.

Hepatic MDA levels were significantly higher in non-OVX rats fed with a HFHF diet as compared with those in non-OVX rats fed with a normal diet (0.14 ± 0.03 vs. 0.12 ± 0.01 nmol/mg protein, respectively; *p* < 0.05). A non-significant increase in hepatic MDA level was also observed in OVX rats with NASH as compared with the OVX control (0.14 ± 0.02 vs. 0.12 ± 0.01 nmol/mg protein, respectively; *p* = 0.051). Genistein treatment did not reduce MDA levels in non-OVX rats with NASH (*p* = 0.055), but it did significantly decrease MDA levels in OVX rats with NASH when compared with the non-treatment group (0.11 ± 0.01 vs. 0.14 ± 0.02 nmol/mg protein, respectively; *p* < 0.01) (Figure 4).

### 3.3. Hepatic Lipid Accumulation

**Figure 5:**
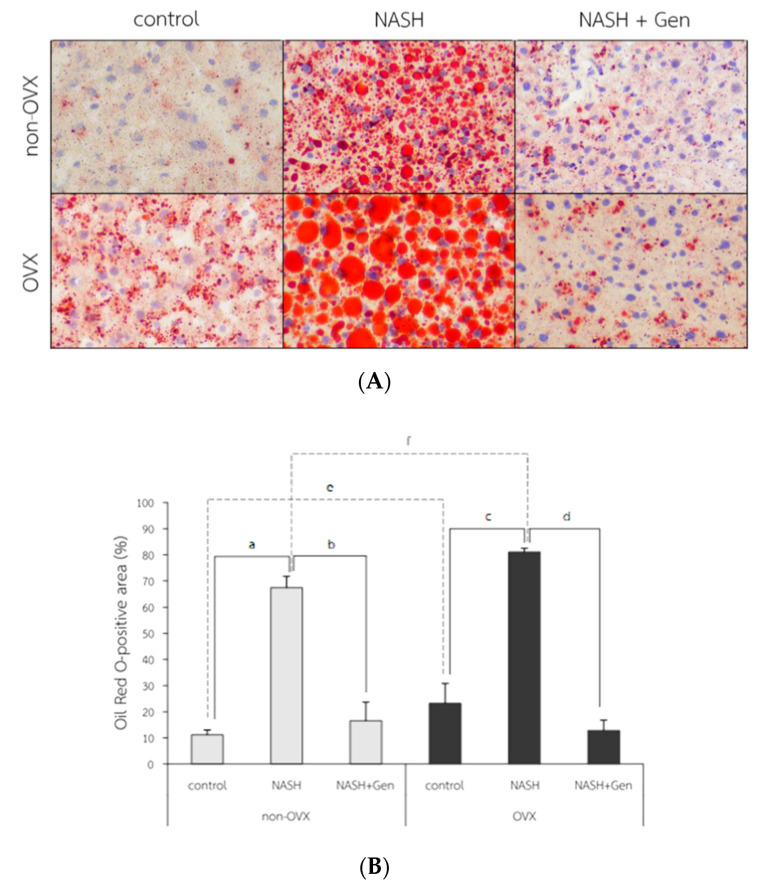
Fat accumulation in liver tissue. (**A**) The representative images of Oil Red O solution (ORO) staining in 6 experimental groups. Original magnification is 400×. (**B**) Percentage of ORO-positive area. a: *p* < 0.05 comparison between control and NASH groups of non-OVX rats. b: *p* < 0.05 comparison between NASH and NASH+Gen groups of non-OVX rats. c: *p* < 0.05 comparison between control and NASH groups of OVX rats. d: *p* < 0.05 comparison between NASH and NASH+Gen groups of OVX rats. e: *p* < 0.05 comparison between control groups of non-OVX and OVX rats. f: *p* < 0.05 comparison between NASH groups of non-OVX and OVX rats.

The Oil Red O (ORO) stain of liver tissue showed increased lipid accumulation in rats fed with HFHF, especially in OVX rats. The attenuation of lipid accumulation was observed after the genistein administration in both non-OVX and OVX rats (Figure 5A). The percentage of ORO-positive area in both non-OVX and OVX rats fed with a HFHF diet were higher than those of control groups (non-OVX rats; 67.42 ± 4.33 vs. 11.17 ± 1.86%, respectively; *p* < 0.01. OVX rats; 81.07 ± 1.44 vs. 23.24 ± 7.63%, respectively; *p* < 0.01). Interestingly, the percentage of ORO-positive area in OVX rats was significantly higher than that in non-OVX rats for both types of diet (normal diet; 23.24 ± 7.63 vs. 11.17 ± 1.86%, respectively; *p* < 0.01. HFHF diet; 81.07 ± 1.44 vs. 67.42 ± 4.33%, respectively; *p* < 0.01). Genistein administration significantly decreased the ORO-positive area in both non-OVX (16.60 ± 7.07 vs. 67.42 ± 4.33%; *p* < 0.01) and OVX rats (12.84 ± 3.96 vs. 81.07 ± 1.44%; *p* < 0.01) as compared with non-treatment groups (Figure 5B).

**Figure 6:**
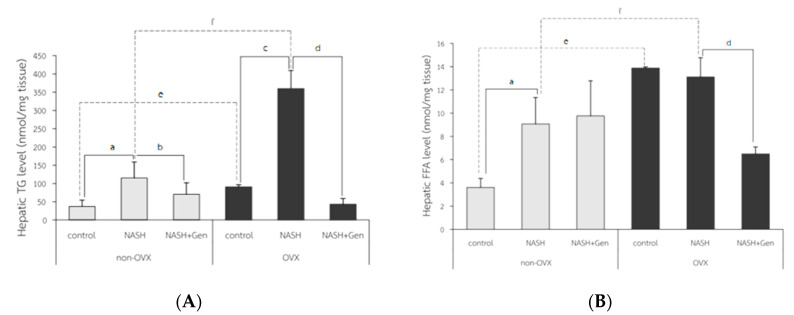
Colorimetry analysis of (**A**) hepatic triglyceride (TG) level and (**B**) hepatic free fatty acid (FFA) level. a: *p* < 0.05 comparison between control and NASH groups of non-OVX rats. b: *p* < 0.05 comparison between NASH and NASH+Gen groups of non-OVX rats. c: *p* < 0.05 comparison between control and NASH groups of OVX rats. d: *p* < 0.05 comparison between NASH and NASH+Gen groups of OVX rats. e: *p* < 0.05 comparison between control groups of non-OVX and OVX rats. f: *p* < 0.05 comparison between NASH groups of non-OVX and OVX rats.

Hepatic triglyceride (TG) levels significantly increased in both non-OVX and OVX rats fed with a HFHF diet as compared with control groups (non-OVX rats; 115.28 ± 43.31 vs. 37.30 ± 17.07 nmol/mg tissue, respectively; *p* < 0.01. OVX rats; 360.12 ± 49.06 vs. 90.75 ± 5.60 nmol/mg tissue, respectively; *p* < 0.01). Similar to the percentage of the ORO-positive area, hepatic TG levels were higher in OVX rats than non-OVX rats of the same diet condition (normal diet; 90.75 ± 5.60 vs. 37.30 ± 17.07 nmol/mg tissue, respectively; *p* < 0.01. HFHF diet; 360.12 ± 49.06 vs. 115.28 ± 43.31 nmol/mg tissue, respectively; *p* < 0.01). Genistein administration significantly lowered hepatic TG levels in both non-OVX and OVX rats when compared with non-treatment groups (non-OVX rats; 70.64 ± 31.29 vs. 115.28 ± 43.31 nmol/mg tissue, respectively; *p* < 0.01. OVX rats; 42.85 ± 15.90 vs. 360.12 ± 49.06, respectively; *p* < 0.01) (Figure 6A).

Hepatic free fatty acid (FFA) levels in OVX rats were significantly higher than those in non-OVX rats for both types of diet (normal diet; 13.89 ± 0.09 vs. 3.62 ± 0.77 nmol/mg tissue, respectively; *p* < 0.01. HFHF diet; 13.11 ± 1.65 vs. 9.07 ± 2.27 nmol/mg tissue, respectively; *p* < 0.01). The HFHF diet significantly increased hepatic FFA levels only in non-OVX rats when compared with the control group (9.07 ± 2.27 vs. 3.62 ± 0.77 nmol/mg tissue, respectively; *p* < 0.01). Genistein significantly reduced hepatic FFA levels only in OVX rats with NASH when compared with the control group (13.11 ± 1.65 vs. 6.50 ± 0.60 nmol/mg tissue, respectively; *p* < 0.01) (Figure 6B).

### 3.4. Hepatocyte Apoptosis by TUNELs

**Figure 7:**
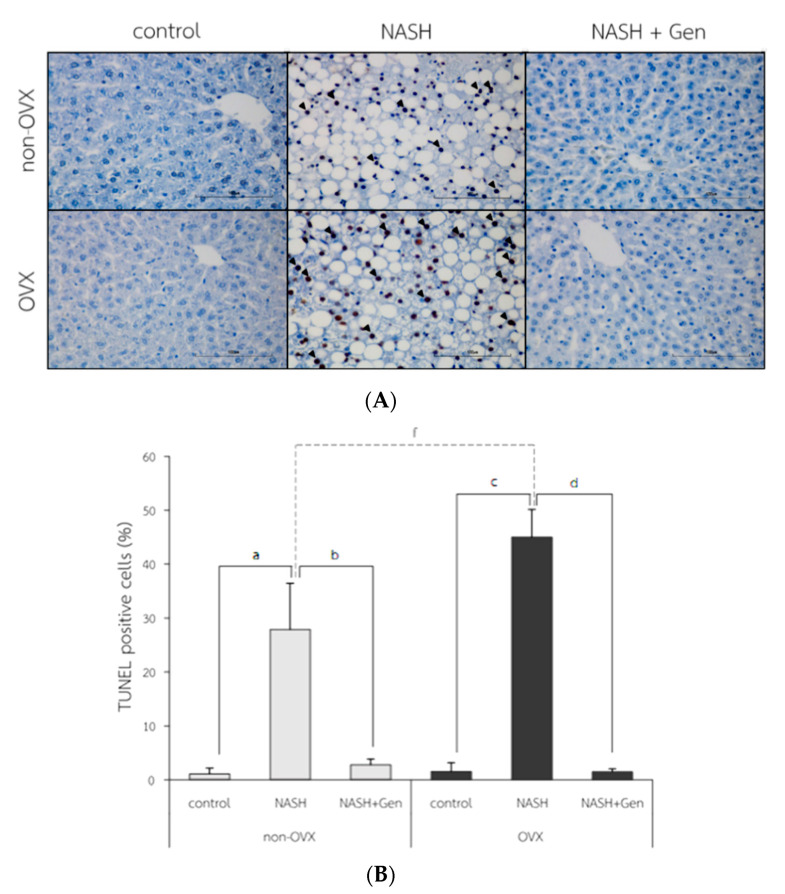
TUNEL positive cells in liver tissue. (**A**) the representative images of hepatocyte apoptosis in liver sections. Arrows indicate TUNEL positive cells. The original magnification is 400×. (**B**) The percentage of TUNEL positive cells. a: *p* < 0.05 comparison between control and NASH groups of non-OVX rats. b: *p* < 0.05 comparison between NASH and NASH+Gen groups of non-OVX rats. c: *p* < 0.05 comparison between control and NASH groups of OVX rats. d: *p* < 0.05 comparison between NASH and NASH+Gen groups of OVX rats. f: *p* < 0.05 comparison between NASH groups of non-OVX and OVX rats.

The TUNEL positive cells were observed with the highest numbers in OVX rats fed with HFHF (Figure 7A). Percentages of TUNEL positive cells were higher in NASH groups than control groups in both non-OVX (27.90 ± 8.55 vs. 1.10 ± 1.06%, respectively; *p* < 0.01) and OVX rats (45.03 ± 5.13 vs. 1.55 ± 1.62%, respectively; *p* < 0.01). The degree of hepatocyte apoptosis was more severe in OVX rats fed with a HFHF diet than that in non-OVX rats (45.03 ± 5.13 vs. 27.90 ± 8.55%, respectively; *p* < 0.01). Genistein administration attenuated the severity of hepatocyte apoptosis as evidenced by the reduction in TUNEL positive cells in both non-OVX and OVX rats when compared with non-treatment groups (non-OVX rats; 2.80 ± 1.07 vs. 27.90 ± 8.55%, respectively; *p* < 0.01. OVX rats; 1.50 ± 0.56 vs. 45.03 ± 5.13%, respectively; *p* < 0.01) (Figure 7B).

### 3.5. Hepatic PPARγ Expression by Western Blot Analysis

**Figure 8:**
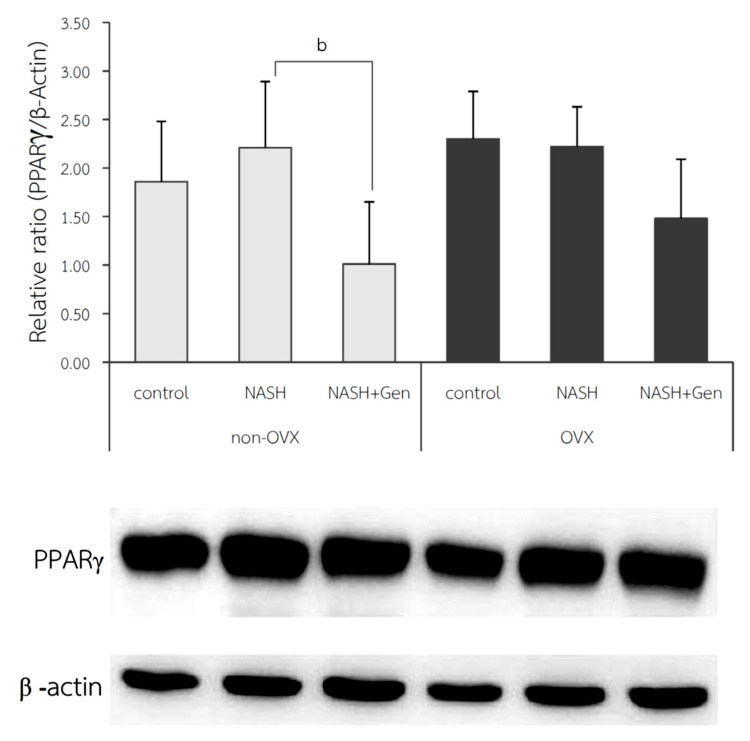
Western blot analysis of PPARγ protein expression in liver tissue. b: *p* < 0.05 comparison between NASH and NASH+Gen groups of non-OVX rats.

The protein expression of PPARγ in liver tissue; shown as relative ratio, was not different between NASH and control groups in both non-OVX (2.21 ± 0.68 vs. 1.86 ± 0.62, respectively; *p* = 0.410) and OVX rats (2.22 ± 0.41 vs. 2.30 ± 0.49, respectively; *p* = 0.834). Genistein treatment could significantly reduce hepatic protein expression of PPARγ only in non-OVX rats (NASH vs. NASH+Gen; 1.01 ± 0.64 vs. 2.21 ± 0.68, respectively; *p* < 0.01). We also observed a non-significant trend toward reduction in hepatic PPARγ protein expression in the genistein-treated OVX rats when compared with the non-treatment group (Figure 8).

### 3.6. Hepatic Adiponectin Expression by Western Blot Analysis

**Figure 9:**
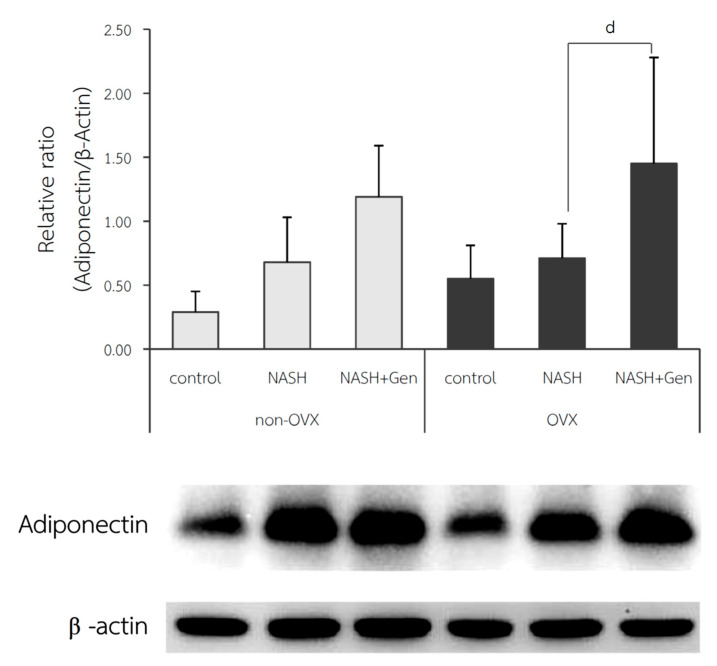
Western blot analysis of adiponectin protein expression in liver tissue. d: *p* < 0.05 comparison between NASH and NASH+Gen groups of OVX rats.

The relative ratio of adiponectin expression in the liver was not different between control and NASH groups in both non-OVX (0.29 ± 0.16 vs. 0.68 ± 0.35, respectively; *p* = 0.215) and OVX rats (0.55 ± 0.26 vs. 0.71 ± 0.27, respectively; *p* = 0.604). A significant increase in adiponectin expression in the liver after genistein treatment was observed only in the OVX rats (NASH+Gen vs. NASH; 1.45 ± 0.83 vs. 0.71 ± 0.27, respectively; *p* < 0.05) but not in the non-OVX ones (NASH+Gen vs. NASH; 11.19 ± 0.40 vs. 0.68 ± 0.35, respectively; *p* = 0.120) (Figure 9).

## 4. Discussion

### 4.1. High-Fat High-Fructose Diet and the Pathogenesis of NASH

Our study demonstrated that NASH could be induced by a HFHF diet in both non-OVX and OVX rats as evidenced by the presence of steatosis, lobular inflammation, and hepatocellular ballooning on histology, and the increases in hepatic triglyceride and free fatty acid levels. Furthermore, oxidative stress (MDA levels) and hepatocyte apoptosis were observed in non-OVX and OVX rats fed with the HFHF diet. High fructose consumption has been implicated in the development of NASH through the activation of de novo lipogenesis and the inhibition of lipid oxidation [16]. In fact, an animal study showed that hepatic steatosis was less severe in fructokinase knock-out mice fed with the HFHF diet as compared with wild-type mice [17]. High fructose influx to the liver from diet consumption contributes to the induction of oxidative stress through the depletion of intracellular ATP, the production of reactive oxygen species and lipotoxicity. Lipid accumulation as a result of fructose metabolism then stimulates the release of pro-inflammatory cytokines from Kupffer cells leading to the development of NASH [18].

In accordance with our findings, a previous animal study reported the increases in hepatic MDA and TNF-α levels in rats with high fat diet induced NASH [11]. Similarly, a clinical study showed that patients with NASH had higher levels of MDA and nitric oxide compared with healthy individuals [19].

Our results demonstrated that a HFHF diet induced significant hepatocyte apoptosis in both non-OVX and OVX rats. Lipotoxicity is the key factor that triggers hepatocyte apoptosis, since fatty acids are mediators of the apoptotic cell death signaling pathway [20]. Previous studies have shown that the exposure of free fatty acids to human liver up-regulates the Fas protein, which is involved in the extrinsic pathway of hepatocyte apoptosis [21]. In addition, inflammatory mediators, especially TNF-α, that are released in response to lipid accumulation can also stimulate the binding of Fas-associated proteins with death domain (FADD) resulting in caspase 8 activation [22]. Moreover, the TNF-α signaling pathway is associated with c-Jun N-terminal kinase (JNK) activation, which also contributes to the development of hepatocyte apoptosis [23]. 

### 4.2. Estrogen Deficiency Exacerbated the Pathogenesis of NASH

Gender differences in the liver response to hepatic insults have been observed in several liver conditions and NAFLD is no exception [24]. Animal models and epidemiologic studies have shown the influence of gender and reproductive states on the severity of NASH. A cross-sectional study showed that men with NAFLD were at greater risk of advanced fibrosis than pre-menopausal women, but the risk of fibrosis was similar between men and post-menopausal women. Moreover, estrogen replacement therapy appeared to reduce the risk of advanced fibrosis in post-menopausal women [8]. These findings were confirmed by another study that the post-menopausal state was independently associated with advanced fibrosis even in non-obese NAFLD patients [25]. In agreement with the clinical studies, our results showed that OVX rats fed with a HFHF diet had higher NASH activity scores, hepatic fat accumulation (based on ORO, hepatic TG, and FFA levels), and hepatocyte apoptosis than the non-OVX counterparts. Moreover, the degree of hepatocyte ballooning, lobular inflammation, and liver fat accumulation were more severe in OVX rats fed with standard diet than non-OVX ones. Our findings suggested that, even in the absence of nutritional factors, estrogen deficiency aggravated NASH. 

Estrogen is involved in many biological activities, including lipid metabolism and anti-inflammation. In fact, a significant number of sex-biased genes are enriched at loci related to lipid metabolism and cardiovascular disease [26]. In the liver of mice fed with a high-fat diet, estrogen (E2) treatment suppressed the expression of a key lipogenic gene, stearoyl-CoA desaturase 1, which was accompanied by the reduction of triglyceride accumulation in liver [27]. Moreover, treatment with estrogen (E2) and estrogen receptor alpha (ERα) agonists has been shown to decrease body weight and total cholesterol level in ovariectomized rats fed with a high fat diet [28]. In addition, estrogen therapy decreased the expression of several lipogenic genes, such as acetyl-coenzyme A (CoA) carboxylate-α and -β, sterol regulatory element-binding protein 1c (SREBP-1c), stearoyl-CoA desaturase, lipoprotein lipase, fatty acid synthase, fatty acid desaturase, and peroxisome proliferator-activated receptor (PPARγ) in adipose tissue of postmenopausal women [29]. Lacking the protective effects of estrogen against lipid dysregulation, it is not surprising that estrogen deficiency promotes lipid accumulation in the liver and worsens NASH pathology.

### 4.3. Genistein Ameliorated Liver Injuries in NASH through Its Anti-Lipogenic, Antioxidant, and Anti-Inflammatory Effects

Genistein is a type of soybean isoflavone that can mimic physiological functions of estrogen by binding to both subtypes of estrogen receptors. Previous studies suggested that genistein alleviated hyperlipidemia through the activation of an estrogenic pathway [30,31]. Genistein inhibits lipogensis and stimulates fatty acid β-oxidation likely through the activation of an adenosine monophosphate-activated protein kinase (AMPK) pathway [32]. In the current study, we found that genistein improved NASH histopathological scores in both OVX and non-OVX rats fed with a HFHF diet. In addition, genistein attenuated hepatic lipid accumulation in both OVX and non-OVX rats, as evidenced by the reduction in the percentage of ORO staining and hepatic TG levels. Hepatic MDA levels, a marker of oxidative stress, also declined in OVX rats that received genistein. The same trend was also observed in non-OVX rats but the changes did not reach statistical significance. Additionally, genistein reduced the degree of hepatocyte apoptosis in genistein-treated rats regardless of the OVX status. 

In line with our results, Jeon and colleagues evaluated the effects of genistein on NAFLD using a model of ApoE knockout mice fed with a high fat diet and found that genistein reduced hepatic steatosis and liver inflammation. The authors showed that genistein acted through the reduction in hepatic TBARS levels, the expression of pro-inflammatory cytokines, hepatic protein levels of PPARγ, and the expression of MGAT1 gene and scavenger receptors; both of which were related to TG synthesis and uptake in the liver [12]. Additionally, another rat study suggested that genistein alleviated NASH through the inhibition of nuclear translocation of NF-κB p65 subunit, and JNK activation leading to the reduction in hepatic inflammatory cytokine levels (i.e., TNF-α, IL-6, TGF-β) and lipid peroxidation markers (i.e., TBARS). Moreover, in a NASH model using mice fed with a methionine-choline-deficient (MCD) diet, genistein reduced the levels of oxidative stress markers (i.e., MDA, heme oxygenase), endoplasmic reticulum stress, and the expression of TNF-α, monocyte chemoattractant protein 1 (MCP-1), toll-like receptor 4 (TLR4), and interleukin 1 beta (IL-1β) [33]. As mentioned previously, TNF-α and JNK activation contribute to the occurrence of hepatocyte apoptosis. The inhibition of JNK activation and TNF-α production by geinstein likely explained the improvement in the degree of hepatocyte apoptosis in geinstein-treated rats in our study. 

### 4.4. Genistein Decreased the Severity of NASH by Down-Regulating Hepatic PPARγ Protein Expression

PPARγ belongs to the ligand-activated nuclear receptor transcription factor superfamily, which plays an important role in lipid storage, glucose metabolism, and adipocyte differentiation. Whether PPARγ protects or aggravates NAFLD remains a matter of debate. Some animal studies have shown that PPARγ overexpression was associated with adipogenic transformation of hepatocytes and the ablation of hepatic PPARγ alleviated hepatic steatosis [34,35]. Moreover, rosiglitazone, a PPARγ agonist, promoted mitochondrial dysfunction, oxidative stress, and liver steatosis in leptin-deficient mice [36]. A human study suggested that PPARγ might promote hepatic steatosis through the up-regulation of the SREBP-1c gene [37]. In contrast to the aforementioned studies, Wu and colleagues demonstrated that PPARγ overexpression was protective against MCD-diet-induced NASH by redistributing fatty acids from the liver to adipose tissue. Moreover, PPARγ activation reduced TNF-α and IL-6 production and increased adiponectin levels [38]. In this study, however, we did not find significant differences in hepatic PPARγ protein levels between control and HFHF diet-fed rats regardless of the OVX status. Interestingly, genistein significantly reduced hepatic PPARγ expression only in non-OVX rats fed with a HFHF diet. 

Recently, genistein has also been shown to be a PPARγ agonist [11,39]. However, our findings do not support genistein as an agonist of PPARγ. Our results are in agreement with several previous studies which demonstrated the suppressive effect of genistein on PPARγ protein expression. The supplementation of genistein in C57BL/6J mice fed with HF diet showed down-regulated expression of lipogenic or adipogenic transcription factors, including PPARγ [40]. Genistein also attenuated hepatic steatosis by reducing the PPARγ target gene monoacylglycerol O-acyltransferase 1, mRNA level in apolipoprotein E-deficient (ApoE(−/−)) mice fed a HF diet [12]. The alleviation effect of genistein on PPARγ expression may be associated with mitogen-activated protein kinase (MAPK) activation [41,42]. MAPK stimulation by genistein leads to PPARγ phosphorylation and suppresses transcriptional activities of PPARγ [43]. With these effects, it can be speculated that genistein is a PPARγ antagonist and protects against NASH [12,40,41,42,43].

### 4.5. Genistein Improved NASH via Up-Regulated Adiponectin Protein Expression in Liver

Adiponectin is an anti-inflammation, anti-diabetic, and anti-atherogenic polypeptide that is released by adipocytes. This kind of adipokine is highly correlated with insulin sensitivity [44]. Epidemiology studies have revealed the strong association of NASH and obesity, particularly visceral obesity. The serum adiponectin was reported to have a negative correlation with obesity, therefore, its level may be altered in NASH. A comparison between healthy and NASH patients showed 50% lower serum adiponectin in individuals with NASH than in healthy individuals [45]. Moreover, diminishing levels of adiponectin in NASH patients was correlated with the severity of liver inflammation [46]. NASH patients did not only show the declination of adiponectin in serum but also the declination of mRNA expression in liver tissue [47].

In contrast, the results of this study did not exhibit the alteration of hepatic adiponectin protein expression between control and NASH groups in both non-OVX and OVX rats. However, oral administration of genistein increased the expression of adiponectin in NASH livers, especially in OVX rats. The lack of differences in adiponectin levels between control and NASH groups in our study could be explained by the fact that rats in both groups had similar weight. Since we did not perform an experiment using genistein in rats that received standard diets, we cannot state with certainty that genistein would not influence adiponectin expression in the absence of NASH. Previous studies reported that adiponectin has a potential action to improve NASH. In ob/ob mice, adiponectin attenuated steatosis and inflammation [48]. In support of our results, previous studies have shown that genistein could modulate adiponectin levels. In mice fed with a HF diet, dietary intake of genistein reduced hepatic steatosis and adiposity, which was related to the elevation of adiponectin and the reduction of leptin in adipose tissue [40]. Although a previous study suggested that adiponectin expression was induced by PPARγ activation [49], our findings indicated otherwise. Similar to our results, Kim and colleagues demonstrated that genistein down-regulated PPARγ expression and increased adiponectin levels in C57BL/6L mice fed with a HF diet [40]. An in vitro study suggested that genistein increased adiponectin level through the inhibition of TNF-α mediated JNK activation, which subsequently inhibited the down-regulation of adiponectin [50]. It is important to note that in this study, we evaluated the hepatic adiponectin expression and not serum adiponectin levels, which might explain why the findings differed from other studies. According to a study by Kaser et al., no correlation between serum adiponectin levels and hepatic adiponectin expression was found and hepatic adiponectin expression might be controlled by local factors [47]. 

## 5. Conclusions

In conclusion, this study demonstrates that estrogen deficiency is a contributing factor to NASH development. Genistein is a potent phytoestrogen that can improve the pathology of NASH through anti-lipid accumulation, anti-oxidation, and anti-apoptosis properties, together with increments of the protective adipokine adiponectin. Accordingly, genistein is a natural product that has protective effects against NASH and might be useful for NASH treatments, especially in estrogen-deficient individuals.

## Data Availability

The data that support the findings of this study are available from the corresponding author, [D.W.], upon reasonable request.
